# What is medical education research? An analysis and definition of subjects, objectives and types of research based on articles that have undergone a peer review process

**DOI:** 10.3205/zma001806

**Published:** 2026-01-15

**Authors:** Katrin Schüttpelz-Brauns, Achim Schneider, Götz Fabry, Jan Matthes, Monika Himmelbauer, Beatrice Buss, Marianne Giesler

**Affiliations:** 1 Heidelberg University, Medical Faculty Mannheim, Mannheim, Germany; 2 University of Ulm, Medical Faculty, Ulm, Germany; 3 University Freiburg, Medical Faculty, Freiburg i. Brsg., Germany; 4 University of Cologne, Faculty of Medicine, Cologne, Germany; 5 Medical University of Vienna, Vienna, Austria; 6 Bern University of Applied Sciences, Department of Health, Bern, Switzerland; 7 Freiburg i. Brsg., Germany

**Keywords:** medical education research, medical education, basic research, applied research, use-inspired basic research

## Abstract

**Background:**

Medical education research (MER) seeks to contribute to the scientific knowledge in this area and to the further development of educational practice. However, the lack of relevance of the studies conducted in this field has been criticized for years. The present work therefore aims to clarify the tasks and objectives of MER and the nature of research in this area. To this end, the subjects, objectives, and types of research that are common to MER are analyzed and categories for these are developed.

**Method:**

The categories for *research subjects* and *research objectives* were developed iteratively in three phases with multiple rounds based on samples of peer-reviewed articles. Depending on the round, two to six people were involved in the independent categorization, finding mutual consent, and further development of the categories. At the same time, *research types* were defined for MER.

**Results:**

169 articles were assessed. Eleven subject categories and eight categories of research objectives were identified, and four types of research were defined as relevant.

**Discussion:**

The categories found for *research subjects* partly coincide with existing category systems but also broaden them. The *research objectives* identified are more specific than they have been before, which limits the scope for interpretation.

**Conclusion:**

The category systems developed can help to define the *subjects* and *objectives* of medical education research more precisely and to differentiate between the *research types* and their significance. In addition, trends and temporary phenomena can also be depicted using the categories found.

## Zusammenfassung

**Hintergrund:**

Medizinische Ausbildungsforschung (MAF) soll einen Beitrag zum Erkenntnisgewinn sowie zur Weiterentwicklung der Ausbildungspraxis leisten. Seit Jahren wird jedoch die mangelnde Relevanz der in diesem Bereich durchgeführten Studien kritisiert. In der vorliegenden Arbeit sollen daher die Aufgaben und Ziele der MAF sowie die Art und Weise der Forschung in diesem Bereich geklärt werden. Hierzu werden die bisher in der MAF üblichen Gegenstände, Ziele und Typen der Forschung analysiert und Kategorien für diese entwickelt.

**Methode:**

Die Entwicklung der Kategorien für *Gegenstände* und *Forschungsziele* erfolgte iterativ in drei Phasen mit mehreren Durchgängen anhand von Stichproben peer-begutachteter Artikel. Je nach Durchgang waren zwei bis sechs Personen in die unabhängige Kategorisierung, Konsentierung und Weiterentwicklung der Kategorien involviert. Zeitgleich wurden *Forschungstypen* für die MAF definiert.

**Ergebnisse:**

169 Artikel wurden beurteilt. Elf *Gegenstandskategorien* sowie acht Kategorien von Forschungszielen wurden identifiziert und vier als relevant erkannte *Forschungstypen* definiert.

**Diskussion:**

Die gefundenen Kategorien für Gegenstände decken sich z. T. mit vorhandenen Kategoriensysteme, erweitern diese jedoch. Die identifizierten *Forschungsziele* sind spezifischer formuliert als es bisher der Fall war und präzisieren somit den Interpretationsspielraum.

**Schlussfolgerung:**

Die entwickelten Kategoriensysteme können helfen, *Gegenstände* und *Forschungsziele* der Medizinischen Ausbildungsforschung genauer zu definieren sowie die *Forschungstypen* und ihre Bedeutung voneinander abzugrenzen. Zudem sind über die gefundenen Kategorien auch Trends und kurzlebige Phänomene abbildbar.

## 1. Introduction

Research in higher education originated in the United States in the 1920s [1]. In medicine, it became established there from the 1950s under the name *medical education research* [2], [3]. Medical education research was a reaction to various socio-historical factors, such as 

*“...the increasing importance of scientific research, the availability of funds for MER [medical education research], the explosive growth of medical knowledge, and concerns about accountability for, and control of, medical education.” *[4]

For years, international journals have repeatedly criticized the lack of relevance of research in medical education research, which can be summarized as a lack of contribution to science in terms of knowledge gain and a lack of implications for practice. The criticism of the lack of scientific knowledge gain relates, e.g., to irrelevant topics or the lack of generalizability of results, the criticism of the lack of practical implications relates, e.g., to the applicability of the results to practice, but also to the measured outcomes. The criticisms are presented and explained in table 1 (Tab. 1). 

To understand how research projects in medical education research could contribute to scientific knowledge or how to further develop practice, it is worth taking a closer look at medical education research. The first question to ask is what specific topics or subjects are being researched in medical education research (*research subjects*). Also, of relevance is what types of research objectives are pursued (*research objectives*) and what types of research (*research type*) are conducted. These three areas of medical education research are examined in more detail in the following sections.

### 1.1. Subjects of medical education research

Looking at relevant textbooks, the wide range of *subjects* of medical education research becomes apparent [5], [6], [7], [8]. This diversity is also reflected in articles in which the *subjects* of medical education research studies are systematically analyzed and categorized [9], [10]. These categories are not always congruent. Moreover, since each *research subject* can be viewed or researched from different perspectives, a one-dimensional enumeration of the *research subjects* is difficult to achieve. 

Since, to our knowledge, there is no generally accepted classification of *research subjects* in studies of medical education research, a structural model developed in educational psychology [11] could be helpful for future categorization of *subjects* in medical education research. In this model, the *subjects* of educational psychology research are arranged along three dimensions. These are *the functional areas of* research, counselling, prevention, intervention as well as monitoring and evaluation, the *educational career*, which covers the entire lifespan and thus emphasizes lifelong learning, and the *activity levels* (micro, meso, macro) at which the tasks are to be performed. Based on this model, the *subjects* of medical education research such as student selection, learning, teaching, examination, curriculum could be assigned to the dimension of *functional areas*, which could be scientifically analyzed at different levels (individual, group, organization), but also in relation to the* educational career* (undergraduate education, post-graduate education, further education). 

### 1.2. Research objectives of medical education research

In medical education, there are two frameworks that address corresponding* research objectives* of medical education research. According to Cook et al., studies aim to describe (*description*, focus on observation), justify (*justification*, “How does an intervention work?”) or explain (*clarification*, “How does something work?”, “Why does something work?”) [12]. In their *research compass*, Ringsted et al. summarize the* research objectives* under the categories of *modelling, justifying, predicting* and *implementing* [13]. However, both papers focus on the type of data collection or study design to answer these questions. How the categories of *research objectives* were developed is left open. 

### 1.3. Types of research in medical education

Looking at the literature on the classification of *research types* in medical education, there are several researchers who place them on a one-dimensional continuum. For example, Albert, who analyzed the discussions in medical education research from a sociological perspective using the field concept of Pierre Bourdieu, found that medical education research is conducted by two interest groups [14]. One group seeks to expand and deepen the knowledge of the research field (epistemic interest). This interest group represents *basic research*. Studies of this *type of research* aim to fundamentally understand general phenomena and their relationships [13], [15], [16]. The aim is to deepen the knowledge and understanding of learning and training within the framework of medical education research [13]. The focus here is on the question “Why does something work?” [13], [16]. The derivation of the research question must therefore be embedded in the existing knowledge on the topic and, if applicable, in a theoretical framework. The other interest group seeks to solve practical problems in medical education with the help of research [14] (application interest). This group represents *applied research*. Studies of this *type of research* aim to solve problems for practice [17]. These can concern both the effectiveness of measures on learning [18], as well as the effect of measures on society or patients [19], [20], [21], [22]. The relevance of research from the perspective of applied researchers is primarily concerned with the practical implications of research findings for improving medical education [13].

The one-dimensional view with the extremes of epistemic interest vs. application interest also exists in other research disciplines and has been described in detail by Stokes [23]. However, Stokes points out that the objectives of studies should be considered two dimensionally. These dimensions represent the presence or absence of the* quest for fundamental understanding* and *considerations of use*. In his two-dimensional quadrant model, three different *types of research* with different objectives can be distinguished [23]. *Pure basic research* is the study of phenomena in a scientific field in order to understand them. It is driven solely by trying to understand without thought of practical use [23]. *Pure applied research* is aimed at an individual, group- or society-related need or application. This is purely for the sake of application with no claim to gain knowledge [23]. The two-dimensional representation generates a third quadrant in which both *quest for fundamental understanding* and *considerations of use are high*. This quadrant represents *use-inspired basic research*. In this type of research, unknown basic principles are tested, and society-related needs are met [23].

Stokes gives concrete examples for three of the quadrants. Although the fourth quadrant is unnamed, it is by no means empty. Stokes assigns research to this quadrant that is not inspired by the goal of understanding nor by the goal of use, but instead systematically investigates certain phenomena [23], [24]. 

### 1.4. Aim and research questions

Previous attempts to describe medical education research in more detail on the basis of its *subjects, objectives* and *types of research*, in order to ultimately demonstrate the significance of its studies in terms of their contributions to knowledge and implications for practice, has revealed a number of gaps. For example, due to the diversity of topics and subjects, there are no uniform *subject categories* in medical education research. Similarly, there are no concrete categories of *research objectives* that are or should generally be pursued in medical education research. And even if the assignment of studies in medical education research to Stokes’ four possible categories appears to be feasible, there is no precise knowledge of the *type of research* conducted in medical education research. To fill these gaps, this study aims to clarify the *subjects* and *objectives* of medical education research and the *nature of research* in this area. To do this, we need to answer the following three questions:

1. What subjects are researched in medical education?
2. What are the objectives of medical education research?
3. Which types of research listed in Stokes' quadrant model [23] can be derived for medical education research

## 2. Methods

It can be assumed that peer-reviewed medical education research articles reflect the possible range of *research subjects, research objectives* and* types of research* from the perspective of the authors, reviewers and editors. To test this assumption, a method based on document analysis [25] was chosen. In three phases with multiple reviews and samples, selected articles were categorized according to area of *research subject, research objective* and *type of research*. In phase 1 (*inductive approach* [26]), categories were developed for these areas to which the studies could be assigned. Phase 2 (*deductive approach* [26]) was used to revise the categories. In phase 3 (*fine-tuning*), the categories and their explanations were refined. Table 2 (Tab. 2) provides an overview of all phases.

### 2.1. Samples

In total, there were five samples of articles (SP1 to S5) that differed in terms of source as well as inclusion and exclusion criteria.

The samples were drawn independently of each other to be able to cover as broad a field as possible when developing categories or reviewing them. Towards the end of the second phase, it became apparent that a further sampling of additional articles would not have led to any new findings, so the articles from S1 to S5 were transferred to a single file. Duplicate articles were identified using the DOI and removed. Articles were then randomly selected from the resulting file, excluding studies from purely medical journals. A detailed description of the sample selection can be found in attachment 1 . 

### 2.2. Procedure

**Figure 1:**
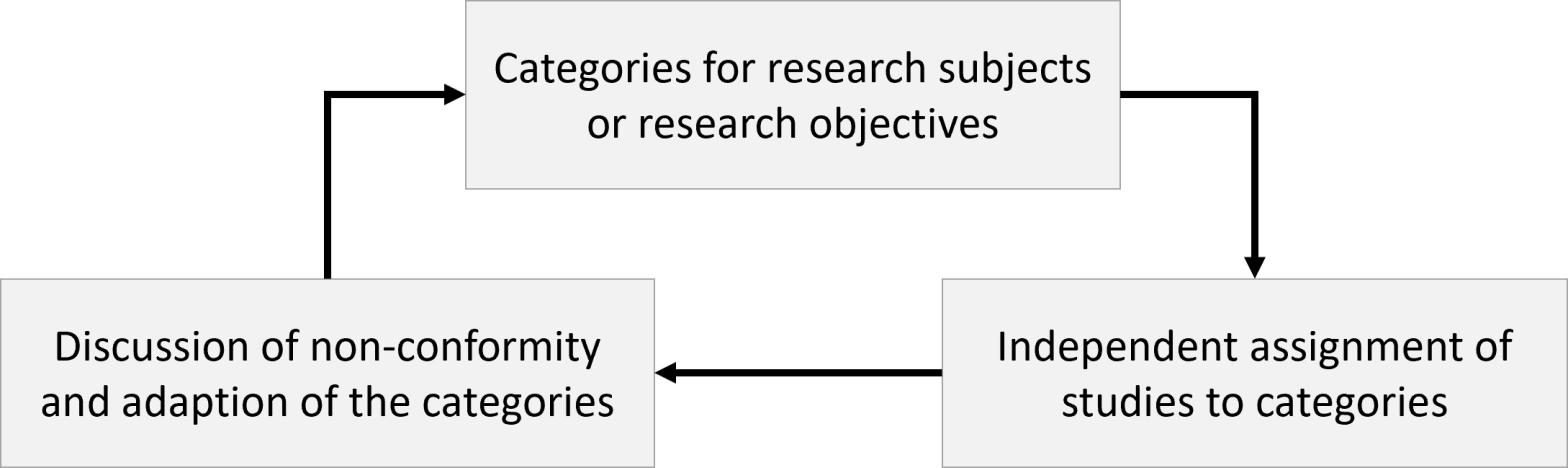
An Iterative approach for the development of categories for subjects and objectives of medical education research

The categories were developed iteratively (see figure 1 (Fig. 1)) over several rounds (see table 2 (Tab. 2)). The categories for *research subjects* and *research objectives* were developed in terms of content, while the categories for the *types of research* according to Stokes [23] only had to be adapted for medical education.

The professional background of four researchers involved in this study is psychology (MG, MH, AS, KSB), two researchers studied medicine (GF, JM) and one physiotherapy (BB). 

#### 2.2.1. Phase 1: Inductive approach (round 1 – round 2)

Round 1 aimed to find initial categories for *research objectives* and to assign articles to the *research types* according to Stokes [23] (see table 2 (Tab. 2)). For this purpose, two authors (MG, KSB) independently paraphrased the *research objective* of the article, the epistemic interest and the application interest. The* research type* was derived from the combination of epistemic and application interest. Discrepancies in the assignment of *research objectives* or *research types* addressed were discussed until agreement was reached. The inductive method was chosen to develop *research objectives* independently of the categories of Cook [12] and Ringsted [13] and, if necessary, to validate these with different methods in the sense of triangulation. 

The aim of round 2 was to review the categories for *research subjects, research objectives* and *research types* by five independent researchers (BB, MH, JM, AS, KSB). The categories for *subjects* and *objectives* as well as the classification into* research types* were predetermined. The structural model of educational psychology described above [11] formed the basis for the development of the initial categories for *subjects* of medical education research. Each dimension of this model was assigned to the study subjects commonly used in medical education research (see table 3 (Tab. 3)). The categories for the *research objectives* were derived from the results of round 1. Since it was assumed that the *research objectives* could be assigned to the categories of the *research types*, the respective assignment of the* research objectives* to the *research types* was predetermined. 

For reasons of research economy, the allocation of articles to be categorized was based on a balanced plan (see attachment 2 ).

Mismatches in the assignment to the categories were identified. If possible, the categories were subsequently reassigned, considering the comments on each *subject* or *objective*.

#### 2.2.2. Phase 2: Deductive approach (round 3 – round 8)

Round 3 to round 5 were used to further develop the categories for *research subjects, research objectives* and classification into *research types* (see table 2 (Tab. 2)). The categories were predefined and derived from the results of the respective previous round. Two researchers (MG, KSB) independently assigned the articles to the categories. Non-matches between the categories were discussed, categories were renamed and reorganized if necessary, and explanations were added to each category. 

The aim of round 6 was to review the categories agreed in round 5, including the explanations for *subjects* and *objectives* as well as the classification of *research types*. For this purpose, five researchers (MG, MH, JM, AS, KSB) independently assessed the articles. Again, for research economic reasons, a balanced plan was used to assign the articles. This plan is equivalent to the one in round 2, only that six articles were assessed in each cell instead of five. 

In both round 7 and round 8, the categories were discussed and adjusted by MG, MH, JM, AS and KSB if they did not match. Prior to round 8, MG and GF respectively analyzed from a psychological and medical point of view, which articles were considered critical, i.e. those articles were reviewed that had an overlap of several *types of research*. The focus was on *research objectives* and their relationship to* research types*.

#### 2.2.3. Phase 3: Fine-tuning (round 9 – round 11)

In the* fine-tuning phase*, the categories and explanations for *research subjects, research objectives* and* research types* were refined (see table 2 (Tab. 2)). Here, too, several rounds took place with discussion of non-agreement, revision resp. refining of the categories and independent assessment, each with three researchers (MG, AS, KSB). 

### 2.3. Analysis of rater agreement

Kappa measures were calculated to determine rater agreement, which are to be regarded as adjusted for chance [27], [28]. Cohen’s kappa (κ) was calculated to determine the agreement of two raters and Fleiss κ to determine the agreement of more than 2 raters.

In phases 1 and 2, Cohen’s κ was calculated for the assessments of the ten and 18 pairs evaluated respectively. These were calculated for all categories of the *research subjects, research objectives* and *research types*. In a second step, the median of these values was determined for all areas as an estimate of the average agreement between all raters, as the median in this case is the measure that best characterizes the entire sample [27]. In phase 1, the results section does not state the significance, as the k values determined are based on the assessments of only five articles. 

In phase 3, Fleiss κ was calculated, as the given items had to be assessed by three researchers. This measure can be used to determine not only the rater agreement of the entire category system, but also for each single category.

According to Landis and Koch, the following values are used to interpret the degree of agreement: κ<0.00 as poor, 0.00-0.20 as slight, 0.21-0.40 as fair, 0.41-0.60 as moderate, 0.61-0.80 as substantial and 0.81-1.00 as almost perfect [28]. In the assessments with three people, the assessment rounds were conducted until sufficient assessment agreement was achieved.

## 3. Results

### 3.1. Sample characteristics

In total, 197 articles were selected. Of these, 28 were included in two samples. Therefore, a total of 169 articles were assessed. During the assessment process, two articles were identified that did not meet the criteria for a study: One article presented a research method and its application in medical education. The other article was a letter to the editor. Both articles were excluded from further analysis. 

Table 4 (Tab. 4) provides information on the articles in each sample. 

### 3.2. Development of the categories

As an iterative approach was adopted in this study, the results of each phase are not only reported in a descriptive manner, as is usual in scientific studies, but are also used to justify the further procedure. 

At the end of phase 1, the following rater agreements were identified for ten rater pairs:

- *Subject areas*
  - *Functional areas category:* κ=-0.111-0.737; κ_Md_=0.143
  - *Educational career category:* κ=-0.250-1.000; κ_Md_=0.302
  - *Activity levels category*: κ=-0.071-0.412; κ_Md_=0.185
- *Research objectives:* κ=-0.087-0.800; κ_Md_=0.243
- Research types: κ=-0.364-0.444; κ_Md_=0.000.

During the inductive phase (phase 1), it became apparent that the *research subjects* assigned to the dimensions of the structural model of educational research (*functional areas, educational career, activity levels*) sometimes overlapped. For example, the *subjects* teaching, learning and curriculum could not always be clearly distinguished from one another. Additionally, some* subjects* could not be clearly assigned to the dimensions of *activity level* and *educational career.* Based on these overlaps, which may have partly caused the low level of rater-agreements, the concept of three dimensions was abandoned and all *research subjects* were grouped into one subject area. The categories for both the *research subjects* and the *research objectives* were gradually adapted in the consensus procedure in the rounds of phase 2. This concerned the categories themselves as well as the explanations for them. Reaching a consensus proved to be particularly difficult, as the researchers of this study had different ideas about science due to their different professional socialization. Additionally, there was a lack of clarity in the terms as well as missing and overlapping categories. It also became apparent that the dimensions *quest for fundamental understanding* and *considerations of use* in the quadrant model according to Stokes [23] can be interpreted differently. With the help of additional literature [23], [29], [30], [31], [32] and the discussion of specific studies, the *research types* were more clearly distinguished from one another.

For the final round of phase 2, the following Cohen's Kappa were calculated for 18 rater pairs:

- *Research subjects*: κ=-0.053-0.722; κ_Md_=0.214
- *Research objective:* κ=-0.111-0.643; κ_Md_=0.268
- *Research type:* κ=-0.176-1.000; κ_Md_=0.354

While fine-tuning, the fourth quadrant was renamed *background research* as suggested by Bush [33]. This change was accompanied by higher level of rater agreement. At the end of this phase, the rater agreements were as follows: for *research subject κ_Fleiss_*=0.63, p<0.001, for *research objective κ_Fleiss_*=0.52, p<0.001 and for *research type κ_Fleiss_*=0.58, p<0.001. The results for each category are listed in attachment 3 , tables A1-A3.

### 3.3. Subjects, objectives and types of research in medical education research

After completion of the three phases, eleven *research subject* categories, eight categories of* research objectives* and explanations of the *research types* are now available (see table 5 (Tab. 5), table 6 (Tab. 6) and table 7 (Tab. 7)).

## 4. Discussion

The starting point for this study is the often-lamented lack of relevance of medical education research: Medical education research is often criticized for contributing little to the advancement of knowledge in this field or for producing findings that have little practical implication.

To better understand how research projects in medical education research can contribute to science in terms of gaining knowledge or further developing practice, the following questions were asked in this study:

1. What subjects are researched in medical education?
2. What are the objectives of medical education research?
3. Which types of research listed in Stokes' quadrant model [23] can be derived for medical education research?

To answer these questions, research articles that have undergone peer-review were used and analyzed accordingly. This was done in an iterative process in which initial categories of each area were reviewed and adjusted. This process of independently reviewing and adjusting categories alternated until a satisfactory rater agreement was achieved. 

The categories found for the *research subjects* partly correspond to previous categories. For example, Raes et al. [9] use similar categories with a slightly different sorting, but in our case, the teaching-learning methods of Raes et al. are divided into teaching methods and traits, motivation and behavior of learners and teachers. We were able to replicate all, but two of Raes et al.’s seven *research subjects*. In the present categorization, the* research subject* faculty development can be assigned to the category’s curriculum, quality criteria and indicators or positions, functions, roles, careers, depending on the topic. Correspondingly, the subject of social competence can be assigned to the category traits, motivation and behavior of students and teachers. Compared to the *subject categories* described by Rotgans [10], no matches could be found, as the categories we have developed are formulated in a more general way. For example, the *research subjects* listed by Rotgans, which are issues in student assessment, objective structured clinical examination, clinical competence assessment, can be assigned to the category with the heading of measuring instruments. The category system developed for *research subjects* in the present study therefore fits in well into previous categorization attempts but also broadens them and has the advantage that new *research subjects* can also be included in this categorization. 

As far as the *research objectives *of medical education research are concerned, previous classifications leave room for interpretation. Both Cook [12] and Ringsted [13] list categories for *research objectives*. However, they neither explain how these *research objectives* are arrived at nor what they actually mean. Some of the studies we examined can be assigned to Cook’s [12] category *description*. For example, there are studies in which phenomena are described that cannot be explained by current theories. These studies provide a starting point for the further development and empirical testing of theories. On the other hand, other studies, which, for example, describe the spread of certain teaching or examination formats without any theoretical or practical implications, would also fall into the description category according to Cook et al.’s categorization. In the present categorization for *research objectives*, however, such studies are assigned to the category of *presentation of a status quo*. 

In summary, the* research objectives* we have identified can be roughly categorized according to previous classifications. However, they are formulated so specifically that they are more likely to guarantee a clear classification, as the scope for interpretation has been largely restricted. Furthermore, additional *research objectives* were identified which relate to the (further) development and verification of measurement instruments and data analysis methods as well as the development of consensus-based, normative guidelines and recommendations. 

Regarding the* types of research* considered relevant for medical education research, three *types of research* were derived in our study: *use-inspired basic research, pure applied research* and *background research*. However, a category of *pure basic research* could not be identified. Referring to the requirements of Eva and Ringsted, this result appears to be partially consistent, because according to their explanations, medical education research should relate to *use-inspired basic research*, i.e. address both *quest for fundamental understanding* and *considerations of use*. Ringsted writes: *“Research in medical education seeks to deepen the knowledge and understanding of learning, teaching and education. It is neither about solving concrete, local problems nor about providing general, universal solutions”* ([13], p.695). Eva is primarily concerned with understanding phenomena that are of practical relevance (*“identify phenomena of interest (and practical relevance)”... “imperative of understanding”*, [16], p.295). To understand these practically relevant phenomena, we need to examine the processes of learning, teaching and education. Since these processes are not directly observable, testable theories that can explain these processes come to the fore. However, these theories are the subject of* pure basic research* [34]. In medical education research, however, *pure basic research* is still carried out far too rarely. In their systematic review, Tolsgaard et al. assigned only 2% of randomized studies to the Bohr quadrant (=*pure basic research*) [35]. The rarity of the occurrence of such studies may therefore be one reason why no studies were found in the present investigation that can be assigned to *pure basic research*. Due to the importance of this category, as described above, the *research types* were supplemented with the category of *pure basic research*.

### 4.1. Strengths and weaknesses of the study

The strength of our study lies in the fact that previous classifications of *research objectives* have a strong focus on the research methodology. Yet the choice of research methodology should only take place after a relevant research question has been identified. However, research methodology did not play a role in our discussions and classifications, so we were able to identify the* research objectives* regardless of the choice of the “right” method. It should be emphasized that peer-reviewed articles, which were assumed to be representative of relevant research, were subject to a procedure based on document analysis. An iterative approach was used to develop the categories and their explanations, alternating between phases of finding mutual consent and verification of agreement. Additionally, researchers from different knowledge cultures were involved. 

Through the iterative process of sampling, categorization, finding mutual consent, checking of rater agreement, revision of the categorization on further articles, etc., it was possible to develop categories that also withstand trends and time-limited topics. Thus, topics such as artificial intelligence or the COVID-19 pandemic are not literally listed in the categories, but are nevertheless implicitly present, e.g. as teaching or examination format or change in student motivation.

The study also has some weaknesses. Particularly in the *fine-tuning phase* (phase 3), some poorly designed studies complicated the categorization process because, for example,* research subjects* and *research objectives* were not explicitly named or were not stringently addressed in different parts of the articles (e.g. abstract vs. full text). These inconsistencies presumably had a negative effect on the rater agreements. Another problem was the different use of the terms *theory* and *model* in some articles. On the one hand, these terms were used to describe natural processes, which were then tested using empirical data (epistemic meaning). On the other hand, the terms* theory* and *model* were used without claiming to explain or describe behavior and experience. Instead, the aim of these studies was to agree on uniform guidelines (normative meaning). The classification of *research types* was also sometimes affected by shortcomings or ambiguities in the articles. Sometimes *research objectives* were described that could have been assigned to *use-inspired basic research* but could not be because of the lack of theoretical reference. In summary, such flaws can limit the extent of rater agreements.

Another possible weakness of the present study is that some categories were chosen less frequently to categorize the articles. This could be because studies that fit into these categories are actually less common. Furthermore, it cannot be ruled out that our explanations of these categories are still too imprecise. Our approach was focused on the development of the categories and their explanations, not on determining and considering the actual occurrence of the studies in the individual categories. 

## 5. Conclusion

Our category systems can help define *research subjects* and *research objectives* of medical education research more precisely as well as better distinguish between the *research types* and their significance. This should make it possible to formulate relevant research questions and choose an approach in which explanatory theories play a role or in which the (general) problem of practice is in the foreground, depending on the type of research in question. The next step is to select the research methodology in such a way that the research question can be answered with the help of the data collected and analyzed. Additionally, the terms used, and in particular their meaning, should be discussed and agreed upon, so that those involved in future studies mean the same thing and their work can contribute more effectively to the gain of knowledge in medical education and ultimately to the further development of practice. 

## Acknowledgements

We would like to thank Olivia Steiger, cand. med. for her contributions to the literature. 

## Authors’ ORCIDs

- Katrin Schüttpelz-Brauns: [0000-0001-9004-0724]
- Achim Schneider: [0000-0002-8602-8535]
- Götz Fabry: [0000-0002-5393-606X]
- Jan Matthes: [0000-0003-2754-1555]
- Monika Himmelbauer: [0000-0001-5516-1993]
- Beatrice Buss: [0000-0001-7270-382X]
- Marianne Giesler: [0000-0001-9384-2343]

## Competing interests

The authors declare that they have no competing interests. 

## Supplementary Material

Description of the sample selection

Balanced plan of 50 articles that were used in phase 1 in the second round to review the categories found

Assessment agreements at the end of the study

## Figures and Tables

**Table 1:**
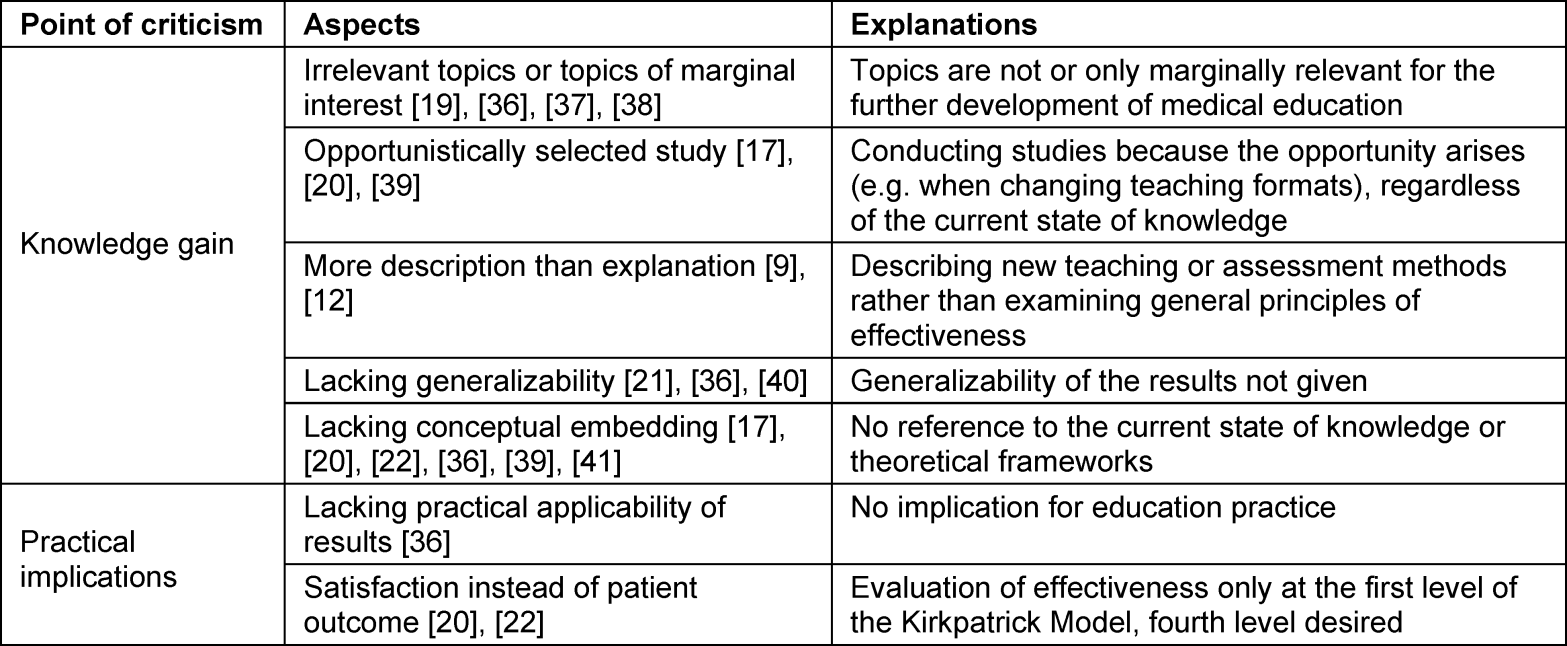
Points of criticism of studies in medical education research

**Table 2:**
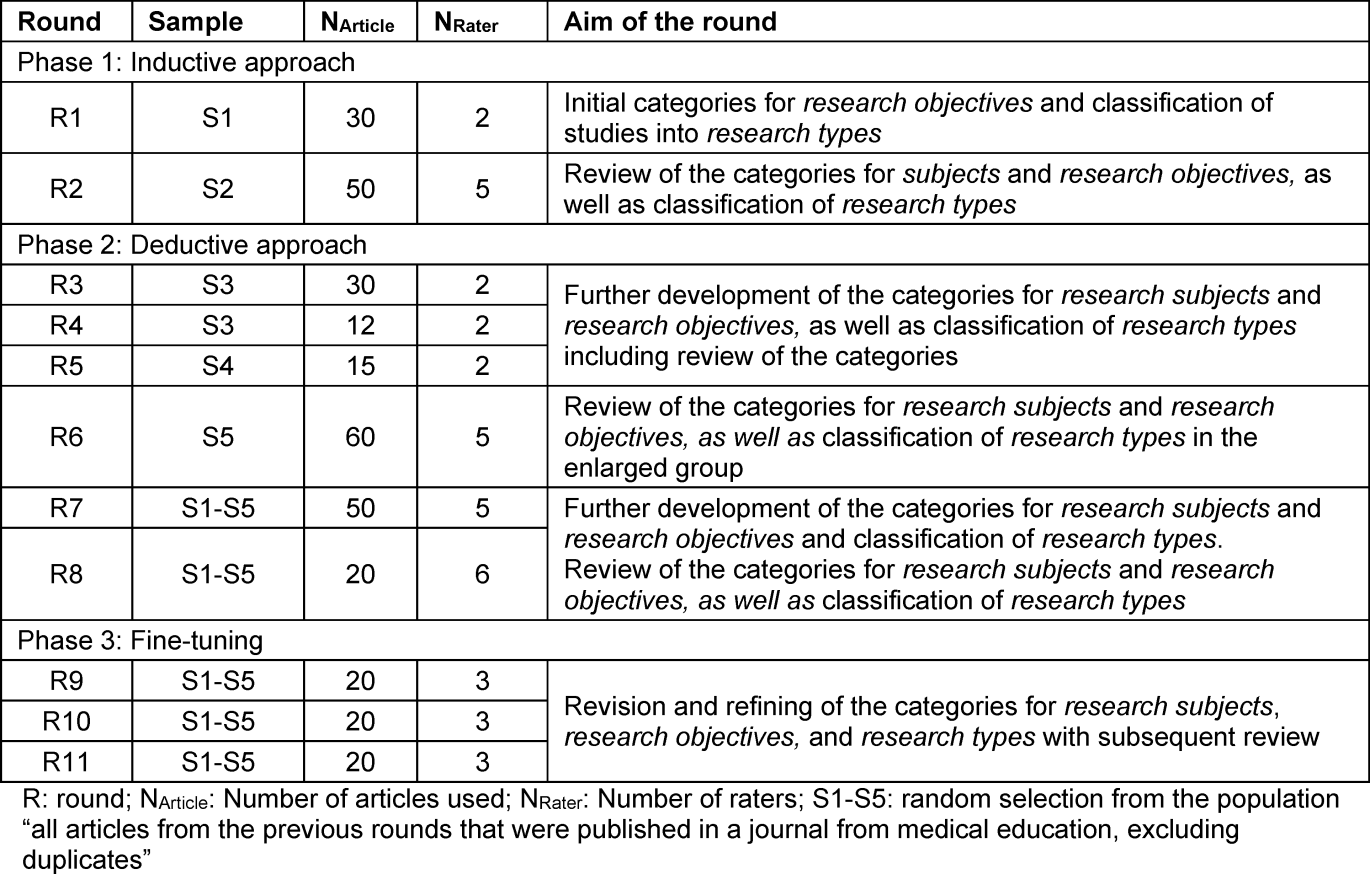
Overview of the phases and rounds of the study

**Table 3:**
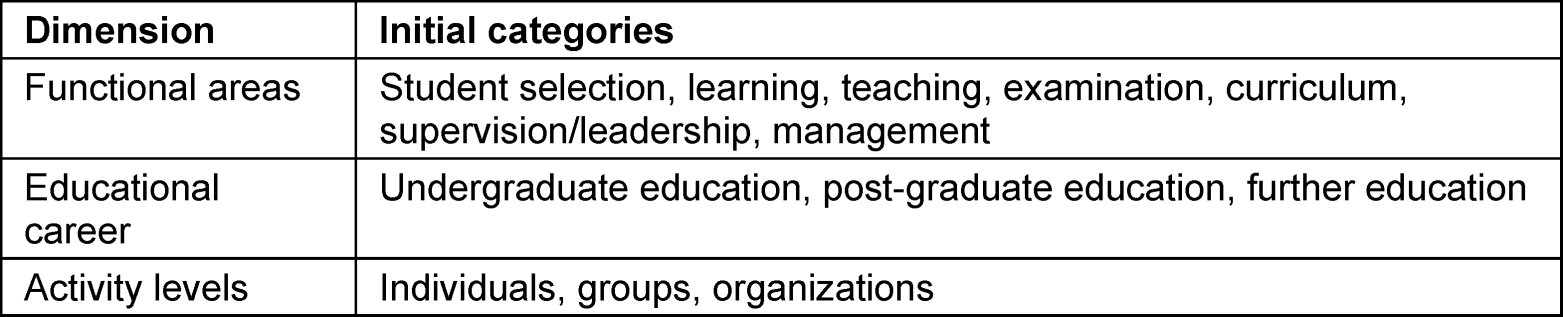
Initial categories for subjects in medical education research

**Table 4:**
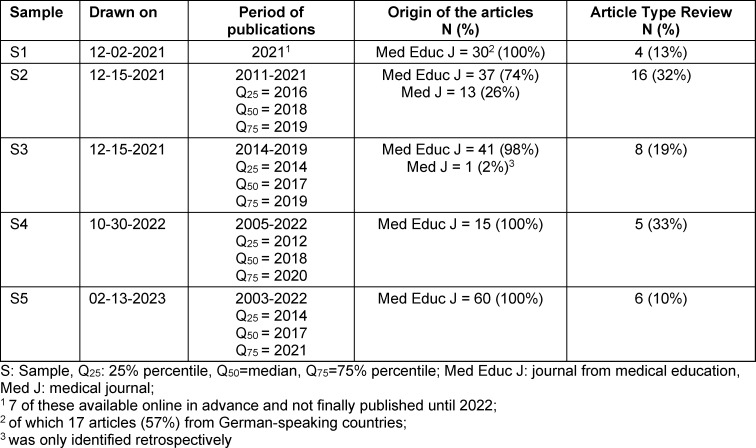
Description of the samples

**Table 5:**
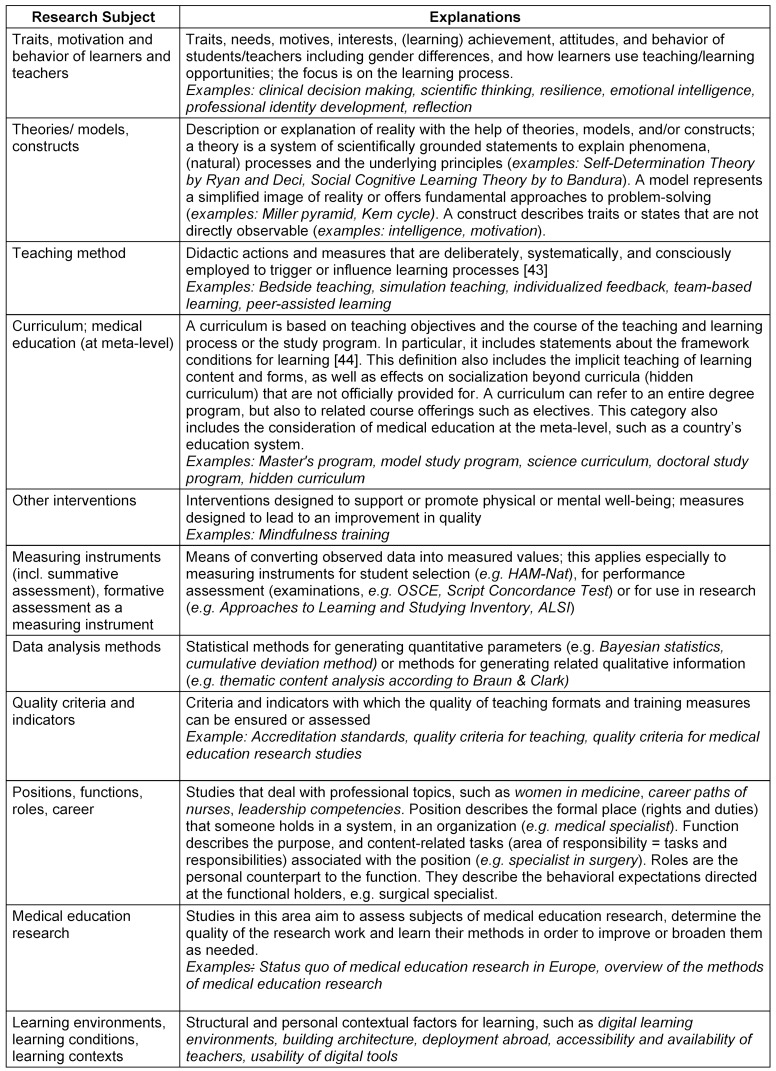
Subjects of medical education research incl. explanations

**Table 6:**
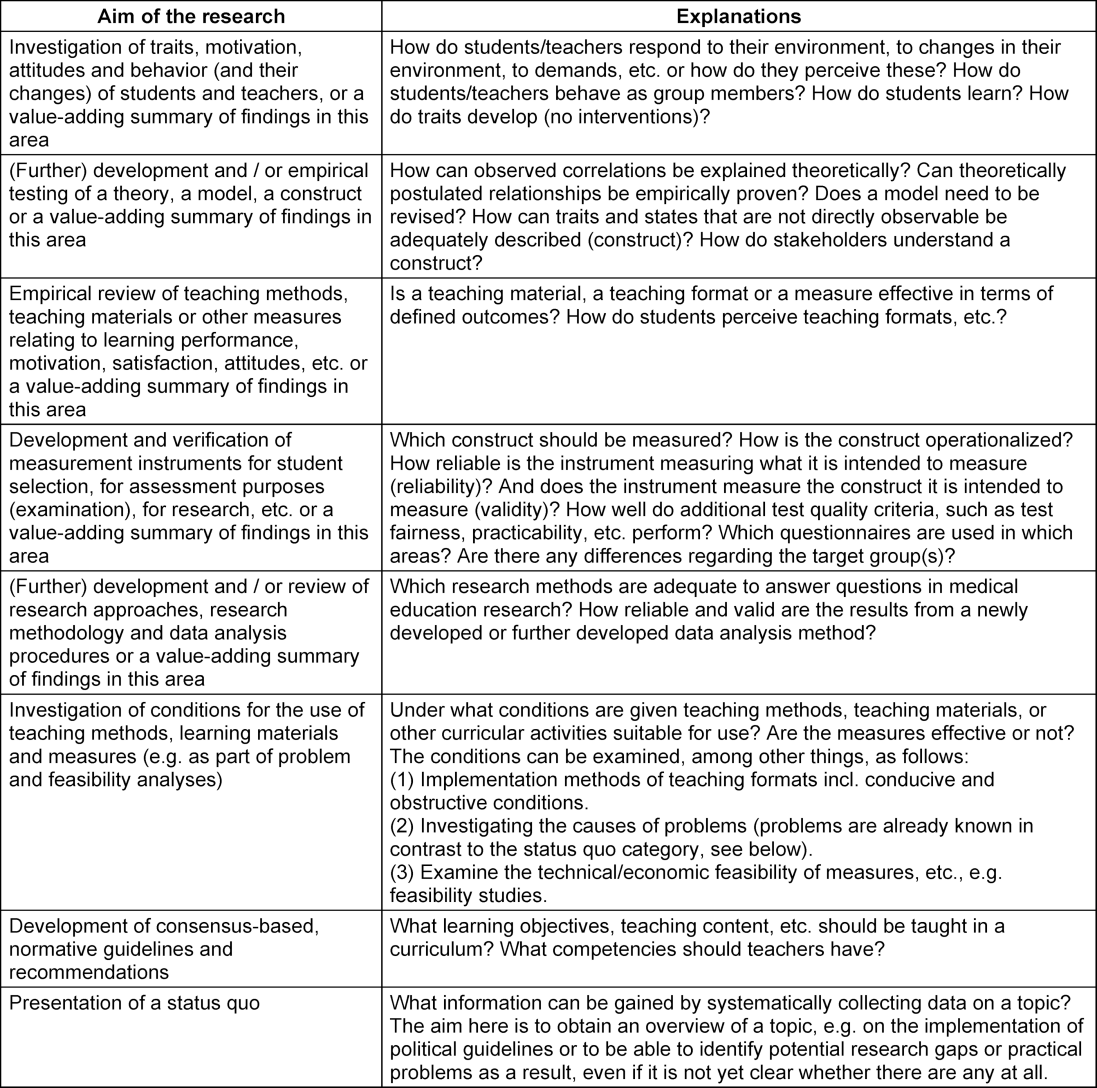
Objectives of medical education research including explanations and examples

**Table 7:**
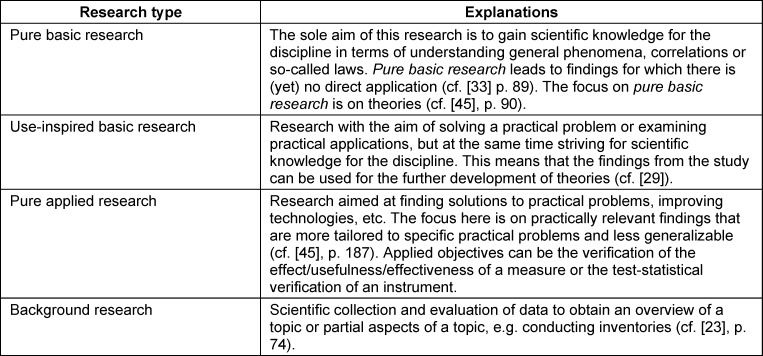
Possible types of research in medical education including explanations
