# Supplementary material for: What is medical education research? An analysis and definition of subjects, objectives and types of research based on articles that have undergone a peer review process
Source: GMS J Med Educ. 2026 Jan 15;43(1):Doc12. doi: 10.3205/zma001806 (PMC12875206; doi:10.3205/zma001806)
Supplement: Description of the sample selection [file JME-43-12-s-001.pdf]

Attachment 1: Description of the sample selection

For the first sample (S1), a total of 30 publications marked as original articles from 2021 from five journals related to medical education research were selected on December 2, 2021. For an initial overview, mainly articles from the GMS *Journal for Medical Education* and only a few articles from journals outside the DACH region (Germany, Austria and Switzerland) were selected.

For the second sample (S2), the focus was extended to the international area and a corresponding Pubmed search was carried out on December 15, 2021, with the search strategy *"medical education" AND study* with a restriction to publications from the last 10 years (period 2011-2021). The 215,249 hits were sorted by *best match*. The following exclusion criteria applied to this sample:

- Articles on methods of medical education research or its challenges/possibilities, etc. (= no studies)
- Non-English articles.

The selection of articles for this and all other samples was based on the order of the hits. Some of the articles came from medical education journals, others from medical journals. As reviewers from medical journals do not necessarily have relevant expertise in medical education research, the selection criteria for the third sample (S3) were more stringent. For this sample, the following inclusion criteria were formulated and specified instead of the exclusion criteria:

- Journal related to healthcare professions and education
- Study (inclusion: systematic collection and analysis of empirical data, both primary and secondary data, approaches: qualitative, quantitative, mixed methods)
- Journal article (exclusion: book, book chapter, conference contribution)

For the fourth sample (S4), a Pubmed search was conducted on October 30, 2022, using the search strategy *"medical education" AND study* with a restriction to publications from the last 22 years (period 2000-2022). The 298,823 hits were sorted by *best match*. The articles were included according to the same inclusion criteria as S3.

Since the fourth sample contained a disproportionately large number of reviews, a further Pubmed search was conducted on February 13, 2023, using the same search algorithm, but additional exclusion criteria were introduced for the sample (S5)

- Article available in S4
- Review article or meta-analysis (summary of the results of several studies).
