# Supplementary material for: What is medical education research? An analysis and definition of subjects, objectives and types of research based on articles that have undergone a peer review process
Source: GMS J Med Educ. 2026 Jan 15;43(1):Doc12. doi: 10.3205/zma001806 (PMC12875206; doi:10.3205/zma001806)
Supplement: Balanced plan of 50 articles that were used in phase 1 in the second round to review the categories found [file JME-43-12-s-002.pdf]

Attachment 2: Balanced plan of 50 articles that were used in phase 1 in the second round to review the categories found

	Rev1	Rev2	Rev3	Rev4	Rev5
Rev1		1-5	6-10	11-15	16-20
Rev2	1-5		21-25	26-30	31-35
Rev3	6-10	21-25		36-40	41-45
Rev4	11-15	26-30	36-40		46-50
Rev5	16-20	31-35	41-45	46-50	

Note: Rev1 to Rev5: assessors; numbers in the cells are the unique identification numbers of the 50 assessed articles

Attachment 2 to Schüttpelz-Brauns K, Schneider A, Fabry G, Matthes J, Himmelbauer M, Buss B. Giesler M. *What is medical education research? An analysis and definition of subjects, objectives and types of research based on articles that have undergone a peer review process*. GMS J Med Educ. 2026;43(1):Doc12. DOI: 10.3205/zma001806
