# Supplementary material for: What is medical education research? An analysis and definition of subjects, objectives and types of research based on articles that have undergone a peer review process
Source: GMS J Med Educ. 2026 Jan 15;43(1):Doc12. doi: 10.3205/zma001806 (PMC12875206; doi:10.3205/zma001806)
Supplement: Assessment agreements at the end of the study [file JME-43-12-s-003.pdf]

Attachment 3: Assessment agreements at the end of the study

Table A1: Agreement for each category for subjects

				Asymptotic		Asymptotic 95% confidence interval	
Evaluation category	CP	κ_{Fleiss}	SE	z	p	Lower limit	Upper limit
1 Traits, motivation and behavior of learners and teachers	0.000	-0.017	0.129	-0.131	0.896	-0.270	0.236
2 Theories/ models, constructs	0.750	0.732	0.129	5.671	0.000	0.479	0.985
3 Teaching method	0.625	0.489	0.129	3.785	0.000	0.236	0.742
4 Curriculum; medical education (at meta-level)	0.833	0.762	0.129	5.902	0.000	0.509	1.015
5 Other interventions	0.500	0.483	0.129	3.739	0.000	0.230	0.736
6 Measuring instruments (incl. summative assessment), formative assessment as measuring instrument	0.750	0.732	0.129	5.671	0.000	0.479	0.985
7 Data analysis methods	0.000	-0.017	0.129	-0.131	0.896	-0.270	0.236
10 Medical education research	0.857	0.838	0.129	6.493	0.000	0.585	1.091
11 Learning environments, learning conditions, learning contexts	0.571	0.515	0.129	3.988	0.000	0.262	0.768

Note: The sample data contains 20 effective subjects and 3 raters. Non-rated categories are not listed. Abbreviations: CP: Conditional probability, κ_{Fleiss}: Fleiss Kappa, SE: Standard error, z: Test statistic, p: Probability of error

Attachment 3 to Schüttpelz-Brauns K, Schneider A, Fabry G, Matthes J, Himmelbauer M, Buss B. Giesler M. *What is medical education research? An analysis and definition of subjects, objectives and types of research based on articles that have undergone a peer review process*. GMS J Med Educ. 2026;43(1):Doc12. DOI: 10.3205/zma001806

Table A2: Agreement for each category for research objectives

			Asymptotic			Asymptotic 95% confidence interval	
Evaluation category	CP	κ_{Fleiss}	SE	z	p	Lower limit	Upper limit
1 Investigation of traits, motivation, attitudes and behavior (and their changes) of students and teachers or a value-adding summary of findings in this area	0.000	-0.017	0.129	-0.131	0.896	-0.270	0.236
2 (Further) development and / or empirical testing of a theory, a model, a construct or a value-adding summary of findings in this area	0.000	-0.034	0.129	-0.267	0.789	-0.288	0.219
3 Empirical review of teaching methods, teaching materials or other measures relating to learning performance, motivation, satisfaction, attitudes, etc. or a value-adding summary of findings in this area	0.800	0.760	0.129	5.887	0.000	0.507	1.013
4 Development and verification of measurement instruments for student selection, for assessment purposes (examination), for research, etc. or a value-adding summary of findings in this area	0.000	-0.034	0.129	-0.267	0.789	-0.288	0.219
5 (Further) development and / or review of research approaches, research methodology and data analysis procedures or a value-adding summary of findings in this area	0.000	-0.017	0.129	-0.131	0.896	-0.270	0.236
6 Investigation of conditions for the use of teaching methods, learning materials and measures (e.g. as part of problem and feasibility analyses)	0.500	0.375	0.129	2.905	0.004	0.122	0.628
7 Development of consensus-based, normative guidelines and recommendations	0.000	-0.017	0.129	-0.131	0.896	-0.270	0.236
8 Presentation of a status quo	0.871	0.733	0.129	5.678	0.000	0.480	0.986

Note: The sample data contains 20 effective subjects and 3 raters. Non-rated categories are not listed.
Abbreviations: CP: Conditional probability, κ_{Fleiss}: Fleiss Kappa, SE: Standard error, z: Test statistic, p: Probability of error

Table A3: Agreement for each category for research types

Evaluation category	CP	κ_{Fleiss}	Asymptotic SE	z	p	Asymptotic 95% confidence interval Lower limit	Upper limit
2 Use-inspired basic research	0.000	-0.071	0.129	-0.553	0.580	-0.324	0.182
3 Pure applied research	0.760	0.589	0.129	4.559	0.000	0.336	0.842
4 Background research	0.871	0.733	0.129	5.678	0.000	0.480	0.986

Note: The sample data contains 20 effective subjects and 3 raters. Non-rated categories are not listed. Abbreviations: CP: Conditional probability, κ_{Fleiss}: Fleiss Kappa, SE: Standard error, z: Test statistic, p: Probability of error

Attachment 3 to Schüttpelz-Brauns K, Schneider A, Fabry G, Matthes J, Himmelbauer M, Buss B. Giesler M. *What is medical education research? An analysis and definition of subjects, objectives and types of research based on articles that have undergone a peer review process*. GMS J Med Educ. 2026;43(1):Doc12. DOI: 10.3205/zma001806
